# Excess risk of cardiovascular disease and mortality after amputation in type 2 diabetes: a nationwide population study from the Swedish National Diabetes Register

**DOI:** 10.1186/s12933-026-03319-5

**Published:** 2026-08-24

**Authors:** Karin Bergqvist, Henrik Imberg, Sara Hallström, Jens Michelsen, Hanna Liljebäck, Stefan Franzén, Anna Norhammar, Annika Rosengren, Marcus Lind

**Affiliations:** 1 Department of Infectious Diseases, Institute of Biomedicine, University of Gothenburg, Gothenburg, Sweden; 2 Department of Molecular and Clinical Medicine, Institute of Medicine, University of Gothenburg, Gothenburg, Sweden; 3 Statistiska Konsultgruppen Sweden AB, Gothenburg, Sweden; 4 Department of Medicine, Geriatrics and Emergency care, Sahlgrenska University Hospital, Region Västra Götaland, Gothenburg, Sweden; 5 Department of Medicine, Skaraborg Hospital, Skövde, Sweden; 6 School of Public Health and Community Medicine, Institute of Medicine, Sahlgrenska Academy, University of Gothenburg, Gothenburg, Sweden; 7 BPM Evidence Statistics, Medical Evidence, BioPharmaceuticals Medical, AstraZeneca, Gothenburg, Sweden; 8 Division of Cardiology, Department of Medicine Solna, Karolinska Institutet and Capio S:t Görans Hospital, Stockholm, Sweden; 9 Department of Medicine, NU-Hospital Group, Trollhättan, Uddevalla, Sweden

**Keywords:** Type 2 diabetes, amputations, cardiovascular risk and mortality

## Abstract

**Background:**

Individuals with diabetes are prone to peripheral angiopathy and neuropathy, predisposing them to diabetic foot ulcers and, ultimately, lower-extremity amputation. The long-term cardiovascular impact of amputation in persons with type 2 diabetes remains insufficiently characterised. The aim of this study was to examine the extent to which lower-extremity amputation in individuals with type 2 diabetes is associated with an increased risk of cardiovascular disease and mortality compared to those with type 2 diabetes without amputation.

**Methods:**

This was an observational, population-based retrospective cohort study with data from the Swedish National Diabetes Register linked to the National Patient Register and the Cause of Death Register. All individuals with type 2 diabetes who underwent a major or minor lower-extremity amputation between 2006 and 2019 were identified and matched to four controls with type 2 diabetes but without previous amputation on age, sex, and calendar time. Adjusted hazard ratios (aHRs) for cardiovascular events and mortality were estimated using cause-specific Cox proportional hazards regression, accounting for demographic and clinical risk factors.

**Results:**

A total of 3,485 individuals with type 2 diabetes who had undergone amputation and 13,940 matched controls without previous amputation were included. Individuals undergoing amputation had a markedly increased risk of cardiovascular disease and mortality, with aHRs (95% CI) of 2.46 (2.31–2.61) for all-cause mortality, 2.43 (2.18–2.71) for cardiovascular mortality, 2.13 (1.92–2.36) for heart failure, 1.79 (1.55–2.08) for myocardial infarction, and 1.52 (1.31–1.76) for stroke. The hazard of all-cause mortality was more than threefold higher in the first year after amputation compared with matched controls and remained about 50% higher after five years.

**Conclusions:**

Lower-extremity amputation in individuals with type 2 diabetes is associated with a substantially increased risk of cardiovascular disease and mortality, independent of pre-existing cardiovascular disease and established risk factors. This underlines the need for intensive cardiovascular risk management both before and after amputation to improve long-term outcomes.

**Supplementary Information:**

The online version contains supplementary material available at 10.1186/s12933-026-03319-5.

**Research insight**.

**What is currently known about this topic?**

- Individuals with type 2 diabetes have a high risk of diabetic foot ulcers and subsequent lower-extremity amputation, and mortality after amputation has historically been reported as high.
- Previous studies suggest increased cardiovascular morbidity and mortality after amputation, but many were conducted before modern cardioprotective diabetes therapies and often did not distinguish between diabetes types or amputation levels.
- The long-term cardiovascular impact of amputation in contemporary type 2 diabetes populations remains insufficiently characterised.

**What is the key research question?**

To what extent is lower-extremity amputation in individuals with type 2 diabetes associated with excess cardiovascular disease and mortality compared with individuals with type 2 diabetes who have not undergone amputation?

**What is new?**

- In this nationwide contemporary cohort, lower-extremity amputation was associated with substantially higher risks of all-cause and cardiovascular mortality, myocardial infarction, heart failure, and stroke—even after adjusting for cardiovascular risk factors.
- The excess risk was most pronounced during the first year following amputation and remained elevated for at least ten years.
- Both major and minor amputations conferred significantly increased cardiovascular risk, with major amputations showing the highest mortality.

**How might this study influence clinical practice?**

The findings highlight the need for intensified cardiovascular prevention and systematic long-term monitoring—before and after amputation—in individuals with type 2 diabetes to improve survival and reduce cardiovascular complications.

## Introduction

Diabetes is a growing global health concern. In 2024, an estimated 589 million people were living with the disease, corresponding to a global prevalence of 11.1%, which is projected to rise [1]. Type 2 diabetes accounts for about 96% of all diabetes cases, and the projected rise is largely driven by type 2 diabetes secondary to increasing rates of obesity in children and adults, with a subsequent rise in diabetes-related complications [1]. Among these, lower-extremity amputation is often a necessary intervention in severe cases of diabetic foot ulcers that do not heal, representing one of the most serious complications of diabetes due to a profound impact on physical function, independence, and quality of life [2–4].

The aetiology of diabetic foot ulcers in type 2 diabetes is multifactorial, involving peripheral arterial disease, peripheral neuropathy, mechanical pressure, hyperglycaemia, and impaired local immune response [5–7]. The prevalence of foot ulcers in individuals with type 2 diabetes is estimated to be around 3–13% and the risk of subsequent amputation increases with time from the onset of the initial ulcer to around 9% at 1 year and 22% at 10 years after first ulceration [8, 9]. With modern multidisciplinary care and timely revascularisation, combined with frequent wound debridement, appropriate dressing changes, and off-loading, it is often possible to limit the extent to minor amputations and thereby reduce long-term disability [10]. However, mortality and cardiovascular morbidity after amputation have been reported to be high [11–16].

Over the past decades, new pharmacological treatments for type 2 diabetes have been developed that not only reduce cardiovascular morbidity and mortality but also exert anti-inflammatory and direct vascular effects [17–19]. Yet, many available studies rely on data collected prior to the modern therapeutic era, and often do not distinguish between diabetes type, amputation level, and diabetic foot disease, and sometimes merge diabetes with peripheral arterial disease, treating them as a single category [11–16, 20].

Although diabetic foot ulcers and their local consequences have historically been well studied, the long-term cardiovascular impact and mortality associated with lower-extremity amputation in people with type 2 diabetes in the modern treatment era remain poorly characterised. In Sweden, the healthcare system is primarily publicly funded through taxation, and access to diabetes care is generally high. Nevertheless, cardiovascular disease remains a major cause of mortality among people with type 2 diabetes [21, 22].

This study aimed to evaluate the excess risk of cardiovascular disease and mortality associated with lower-extremity amputation in type 2 diabetes, and to assess whether these associations persist after adjustment for pre-existing cardiovascular disease and conventional risk factors.

## Methods

This was an observational, population-based retrospective cohort study of individuals with type 2 diabetes in Sweden. Data were obtained from the nationwide Swedish National Diabetes Register (NDR) and linked, via unique personal identification numbers, to the Swedish National Patient Register (NPR) and the Cause of Death Register (CDR). The NDR includes detailed clinical information for the vast majority of individuals with type 2 diabetes in Sweden. The NPR and CDR, maintained by the National Board of Health and Welfare, include nationwide data on hospital discharges and causes of death, respectively.

The NDR is a nationwide quality register that includes individuals with diabetes treated in both primary care and specialist outpatient clinics across Sweden [21–23]. Clinical information is recorded by healthcare professionals during routine clinical visits into a standardised web-based system or electronically transferred from patient record systems to the NDR. The NDR includes data on patient characteristics, risk factors for complications such as HbA1c, BMI, blood pressure, blood lipids, smoking, treatments, and information on diabetes-related complications. For research purposes, NDR data can be linked to other nationwide health registers, including the National Patient Register and the Cause of Death Register, using the unique personal identification number assigned to all Swedish residents [21–23]. The NPR includes nationwide data on hospital inpatient care since 1964 and specialist outpatient care since 2001, but does not include primary care data.

### Study cohort

The study cohort was derived from the NDR and included all adults (aged ≥ 18 years) with type 2 diabetes who underwent a lower-extremity amputation between 2006 and 2019. Type 2 diabetes was defined according to registration as clinical type 2 diabetes in the NDR and an epidemiological definition requiring an age at diagnosis > 40 years or absence of insulin treatment at diagnosis.

Amputations were categorised as major (above the ankle) or minor (below the ankle). For each individual undergoing amputation, four controls were randomly selected using risk-set sampling from individuals with type 2 diabetes who were alive, without prior amputation, and at risk at the time of the case’s amputation. Controls were matched on age, sex, and calendar time and were assigned the same index date as the corresponding case. Individuals selected as controls could later become cases. Both cases and controls were required to be free from myocardial infarction, prior hospitalisation for heart failure, and stroke before the index date. For the major-amputation cohort, individuals could have had a prior minor amputation but no previous major amputation.

## Procedures

Amputations were identified in the NPR using diagnosis codes from the International Statistical Classification of Diseases and Related Health Problems, Tenth Revision (ICD-10), in combination with surgical procedure codes from the Swedish Classification of Surgical Procedures (KKÅ). Major amputations, defined as amputations above the ankle, were identified using codes NGQ09, NGQ19, NGQ99, NFQ19, NHQ11, Z89.5, Z89.6, and Z89.7. Minor amputations, defined as amputations below the ankle, were identified using NHQ12, NHQ13, NHQ14, NHQ16, NHQ17, NHQ99, and Z89.4.

## Outcomes

The study outcomes included all-cause mortality, cardiovascular mortality, myocardial infarction, heart failure, and stroke. All-cause and cardiovascular mortality were obtained from the CDR, where cardiovascular mortality was defined using the following ICD-10 codes for an underlying cause of death I20–I25, I61, or I63–I64. Cardiovascular outcomes were identified from the NPR using ICD-10 code I21 for myocardial infarction, I50 for heart failure, and I60–I64 for stroke. In post hoc analyses, we also evaluated 30-day all-cause mortality, defined as death from any cause within 30 days of the index date.

Time to event was calculated from the index date (i.e. the date of amputation for cases or the corresponding matched date for controls) until the first occurrence of the outcome, death, migration, or end of follow-up on 31 December 2019. Risks of all-cause mortality, cardiovascular mortality, myocardial infarction, heart failure, stroke, and 30-day mortality were analysed among individuals with type 2 diabetes, comparing those with any amputation (overall, minor, or major) to those with type 2 diabetes without amputation. In addition, time-dependent hazards of cardiovascular outcomes were examined over a 10-year follow-up period.

## Covariates

Baseline covariates were obtained from the most recent NDR record within two years prior to the index date. Variables included age, sex, smoking status, body mass index (BMI), systolic and diastolic blood pressure, glycated haemoglobin (HbA1c), albuminuria (categorised as normo-, micro-, or macroalbuminuria), estimated glomerular filtration rate, low-density lipoprotein cholesterol, high-density lipoprotein cholesterol, and diabetes duration. eGFR was calculated according to the Chronic Kidney Disease Epidemiology Collaboration equation.

## Statistics

Descriptive statistics were summarised using means and standard deviations (SDs) or medians and interquartile ranges (IQRs) for continuous variables, as appropriate, and counts and percentages for categorical variables. Incidence rates were calculated as events per 1000 person-years with 95% confidence intervals (CIs). The cumulative incidence of all-cause mortality was estimated as one minus the Kaplan–Meier survival function. Cumulative incidence curves for other cardiovascular outcomes were estimated using the Aalen–Johansen estimator, accounting for death as a competing event.

Time-to-event outcomes were analysed using cause-specific Cox proportional hazards regression, with censoring at death for cardiovascular outcomes other than mortality. Results are presented as hazard ratios (HRs) and 95% CIs, comparing individuals with amputation to matched controls. Adjusted hazard ratios (aHRs) were estimated accounting for baseline covariates as described above. Continuous covariates were modelled using restricted cubic splines to allow for non-linear associations.

Thirty-day mortality was analysed by comparing risks between individuals with amputation and matched controls. Risk differences and risk ratios were estimated and are presented with 95% confidence intervals estimated using the Farrington–Manning score method.

Time-dependent effects of amputation were evaluated by allowing the hazard ratio to vary over follow-up time using interaction terms between amputation status and time, modelled with natural cubic splines with knots placed at 1, 5, and 10 years since the index date. In models with time-varying effects, all covariates were specified at baseline and were not updated during follow-up.

Missing baseline covariate data were handled using multiple imputation by chained equations (MICE), under the assumption that data were missing at random. Continuous variables were imputed using linear regression and binary variables using logistic regression. Historical data were incorporated as auxiliary variables to improve imputation accuracy and support the plausibility of the missing-at-random assumption.

All tests were two-tailed and conducted at the 5% significance level. Statistical analyses were conducted using R software, version 4.5.1 (R Foundation for Statistical Computing, Vienna, Austria), and SAS/STAT^®^ software, version 9.4 (SAS Institute Inc., Cary, NC, USA). Cox regression was performed in R using the *survival* package (version 3.8-3), multiple imputation using the *mice* package (version 3.18.0), and non-linear effects were evaluated using the *splines* package (version 4.5.1).

## Results

### Baseline characteristics

Between 2006 and 2019, a total of 3,485 individuals with type 2 diabetes who underwent a first lower-extremity amputation were identified and matched to 13,940 controls without amputation on age, sex, and calendar time (Supplementary Figure S1). The mean age at inclusion was 74 (SD 12) years, and 35% were women. The mean (SD) HbA1c was 63 (19) mmol/mol (7.9 [1.7]%) among those with amputation and 54 (13) mmol/mol (7.1 [1.2]%) among controls. Current smoking was reported by 18% of individuals with amputation and 10% of controls. Microalbuminuria was present in 30% and macroalbuminuria in 18% of those with amputation, compared with 18% and 6% among controls, respectively (Table 1).

**Table 1:** Baseline characteristics of individuals with type 2 diabetes and any lower-extremity amputation compared with age- and sex-matched controls

	Any amputation (*n* = 3485)	Controls (*n* = 13,940)
Age (years)	74 (12)	74 (12)
Female sex, n (%)	1218 (35%)	4872 (35%)
Diabetes duration (years)	17 (10)	12 (8)
*Missing*,* n*	992	4318
Body mass index (kg/m^2^)	28.4 (5.8)	28.7 (5.5)
*Missing*,* n*	965	4,176
Smoking, n (%)	463 (18%)	1014 (10%)
*Missing*,* n*	911	4,043
Systolic blood pressure (mmHg)	138 (19)	137 (16)
*Missing*,* n*	879	3,927
Diastolic blood pressure (mmHg)	74 (11)	75 (9)
*Missing*,* n*	879	3,927
HbA1c (mmol/mol)	63 (19)	54 (13)
HbA1c (%)	7.9 (1.7)	7.1 (1.2)
*Missing*,* n*	878	3,913
Peripheral arterial disease, n (%)	2157 (62%)	773 (5.5%)
Microalbuminuria, n (%)	735 (30%)	1744 (18%)
Macroalbuminuria, n (%)	452 (18%)	588 (6.2%)
*Missing*,* n*	1038	4494
eGFR (mL/min/1.73 m²)	71 (26)	77 (21)
*Missing*,* n*	900	3984
HDL cholesterol (mmol/L)	1.27 (0.44)	1.31 (0.40)
*Missing*,* n*	1097	4654
LDL cholesterol (mmol/L)	2.57 (0.99)	2.61 (0.91)
*Missing*,* n*	1098	4624

Data are presented as mean (SD) or number (%). The number of missing values is shown for each variable, if applicable.

Age was defined as age at amputation for individuals undergoing amputation and at the corresponding index date for controls. Peripheral arterial disease was identified using ICD-10 codes I70.2 and I73.9 recorded in the Swedish National Patient Register before the amputation or index date. All other variables were based on the most recent registration in the Swedish National Diabetes Register prior to the amputation or index date. The mean (SD) interval between NDR registration and the amputation/index date was 1.0 (1.4) years among individuals undergoing amputation and 0.9 (1.3) years among controls.

Abbreviations: eGFR, estimated glomerular filtration rate; HbA1c, glycated haemoglobin; HDL, high-density lipoprotein; LDL, low-density lipoprotein.

The cohort included 2435 individuals with major amputation and 1449 with minor amputation, with corresponding control groups of 9740 and 5796 individuals, respectively. Characteristics of these subcohorts are presented in Supplementary Table S1.

## Mortality and cardiovascular outcomes

**Fig. 1:**
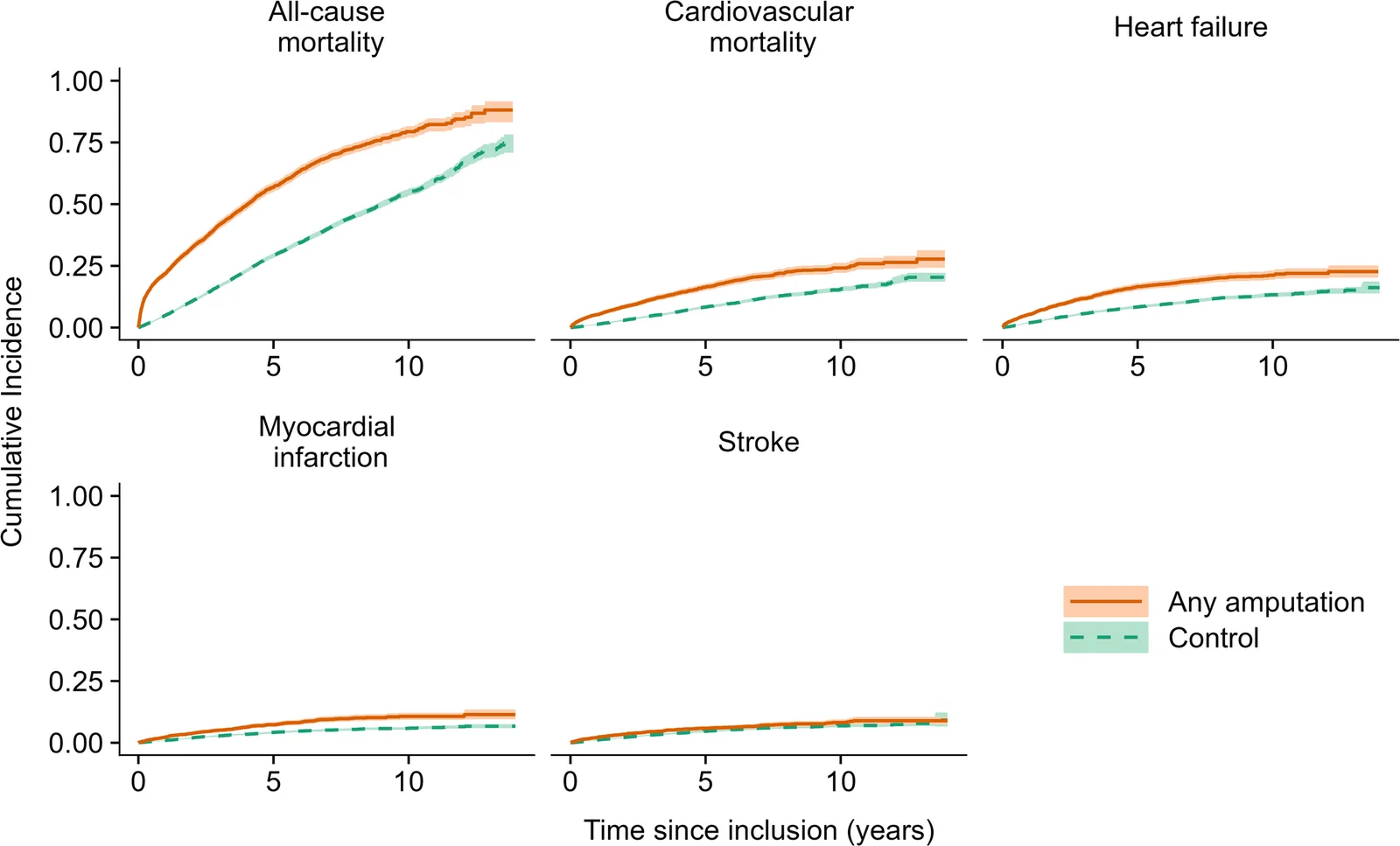
Cumulative incidence curves for all-cause mortality, cardiovascular mortality, myocardial infarction, heart failure, and stroke in individuals with type 2 diabetes with any lower-extremity amputation (solid orange line) and matched controls (dashed green line). All-cause mortality was analysed using the Kaplan–Meier method; other outcomes were analysed using the Aalen–Johansen estimator accounting for death as a competing event. Shaded areas represent 95% confidence intervals

During a median (IQR) follow-up of 3.5 (1.5–6.3) years, there were 1925 deaths (55%) among individuals with amputation and 4,397 deaths (32%) among matched controls (Table 2; Fig. 1). The incidence rate of all-cause mortality was 179.0 per 1000 person-years among individuals with amputation and 71.7 per 1000 person-years among controls, corresponding to an aHR of 2.46 (95% CI 2.31–2.61, *P* < 0.001). Thirty-day mortality was 61.1 versus 3.6 per 1000 persons among individuals with amputation and controls, respectively, corresponding to a risk difference of 57.5 per 1000 individuals (95% CI 53.0–62.1) and a risk ratio of 17.0 (95% CI 12.6–23.1).

**Table 2:** Incidence rates and hazard ratios for all-cause mortality and cardiovascular outcomes in individuals with type 2 diabetes with any lower-extremity amputation compared with age- and sex-matched controls

Outcome	Study group	No. events	Follow-up time (1000 PY)	IR (95% CI) per 1000 PY	Unadjusted HR (95% CI)	*P*	Adjusted HR (95% CI)	*P*
All-cause mortality	Control	4397	61.3	71.7 (69.6, 73.9)				
	Any amputation	1925	10.8	179.0 (171.1, 187.2)	2.53 (2.40, 2.67)	< 0.001	2.46 (2.31, 2.61)	< 0.001
Cardiovascular mortality	Control	1251	61.3	20.4 (19.3, 21.6)				
	Any amputation	564	10.8	52.5 (48.2, 57.0)	2.64 (2.39, 2.92)	< 0.001	2.43 (2.18, 2.71)	< 0.001
Myocardial infarction	Control	880	59.2	14.9 (13.9, 15.9)				
	Any amputation	319	10.2	31.2 (27.8, 34.8)	2.08 (1.83, 2.37)	< 0.001	1.79 (1.55, 2.08)	< 0.001
Heart failure	Control	1655	58.1	28.5 (27.1, 29.9)				
	Any amputation	696	9.6	72.4 (67.1, 78.0)	2.54 (2.33, 2.78)	< 0.001	2.13 (1.92, 2.36)	< 0.001
Stroke	Control	959	59.3	16.2 (15.2, 17.2)				
	Any amputation	269	10.2	26.3 (23.3, 29.6)	1.61 (1.41, 1.85)	< 0.001	1.52 (1.31, 1.76)	< 0.001

Hazard ratios were estimated using Cox proportional hazards regression, unadjusted and adjusted for age, sex, diabetes duration, smoking status, BMI, blood pressure, HbA1c, albuminuria, eGFR, LDL and HDL cholesterol at baseline.

Abbreviations: BMI, body mass index; CI, confidence interval; eGFR, estimated glomerular filtration rate; HbA1c, glycated haemoglobin; HDL, high-density lipoprotein; HR, hazard ratio; IR, incidence rate; LDL, low-density lipoprotein; PY, person-years.

The incidence rate of cardiovascular mortality was 52.5 per 1000 person-years among individuals with amputation and 20.4 per 1000 person-years among controls, corresponding to an aHR of 2.43 (95% CI 2.18–2.71, *P* < 0.001). For other cardiovascular outcomes, the incidence rates were 31.2 versus 14.9 per 1000 person-years for myocardial infarction, 72.4 versus 28.5 for heart failure, and 26.3 versus 16.2 for stroke, with corresponding aHRs of 1.79 (95% CI 1.55–2.08) for myocardial infarction, 2.13 (95% CI 1.92–2.36) for heart failure, and 1.52 (95% CI 1.31–1.76) for stroke (all *P* < 0.001).

### Subgroup analyses of major and minor amputations

**Fig. 2:**
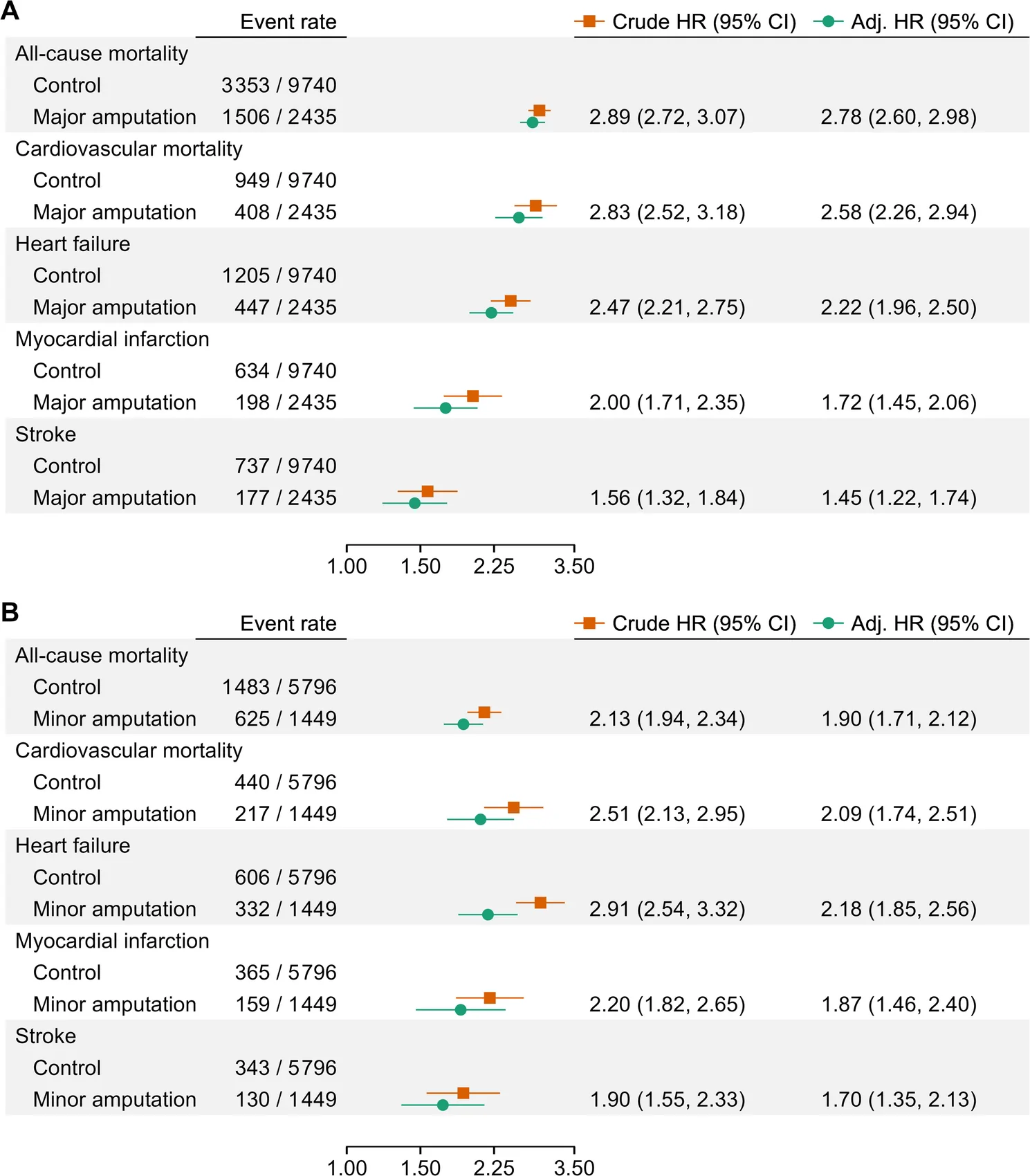
Crude and adjusted hazard ratios (HRs; aHRs) for all-cause mortality, cardiovascular mortality, heart failure, myocardial infarction, and stroke in individuals with type 2 diabetes following (A) major and (B) minor lower-extremity amputation compared with age- and sex-matched controls. Points and error bars represent HRs with 95% confidence intervals (CIs). Adjusted models account for age, sex, smoking status, BMI, blood pressure, HbA1c, albuminuria, eGFR, LDL and HDL cholesterol at baseline

Among individuals with type 2 diabetes who underwent a major amputation (*n* = 2435), the all-cause mortality rate was 197.4 versus 71.1 per 1000 person-years among matched controls (aHR 2.78, 95% CI 2.60–2.98, *P* < 0.001). For cardiovascular mortality, incidence rates were 56.8 versus 20.3 per 1000 person-years (aHR 2.58, 95% CI 2.26–2.94, *P* < 0.001). The risks of other cardiovascular outcomes were also increased among individuals with major amputation compared with matched controls, with aHRs of 2.22 (95% CI 1.96–2.50) for heart failure, 1.72 (95% CI 1.45–2.06) for myocardial infarction, and 1.45 (95% CI 1.22–1.74) for stroke (all *P* < 0.001) (Fig. 2A). Thirty-day mortality was 83.8 versus 5.2 per 1000 individuals in the major amputation and matched control groups, respectively, corresponding to a risk difference of 78.5 per 1000 individuals (95% CI 72.2–84.9) and a risk ratio of 16.0 (95% CI 11.8–21.7).

For minor amputation (*n* = 1449), the aHRs were 1.90 (95% CI 1.71–2.12) for all-cause mortality and 2.09 (95% CI 1.74–2.51) for cardiovascular mortality. For other cardiovascular outcomes, the aHRs were 2.18 (95% CI 1.85–2.56) for heart failure, 1.87 (95% CI 1.46–2.40) for myocardial infarction, and 1.70 (95% CI 1.35–2.13) for stroke (all *P* < 0.001) (Fig. 2B). Thirty-day mortality was 15.2 versus 2.6 per 1000 individuals in the minor amputation and matched control groups, respectively, corresponding to a risk difference of 12.6 per 1000 individuals (95% CI 8.5–16.7) and a risk ratio of 5.9 (95% CI 3.1–11.2).

### Time-dependent hazard ratios for mortality and cardiovascular outcomes

**Fig. 3:**
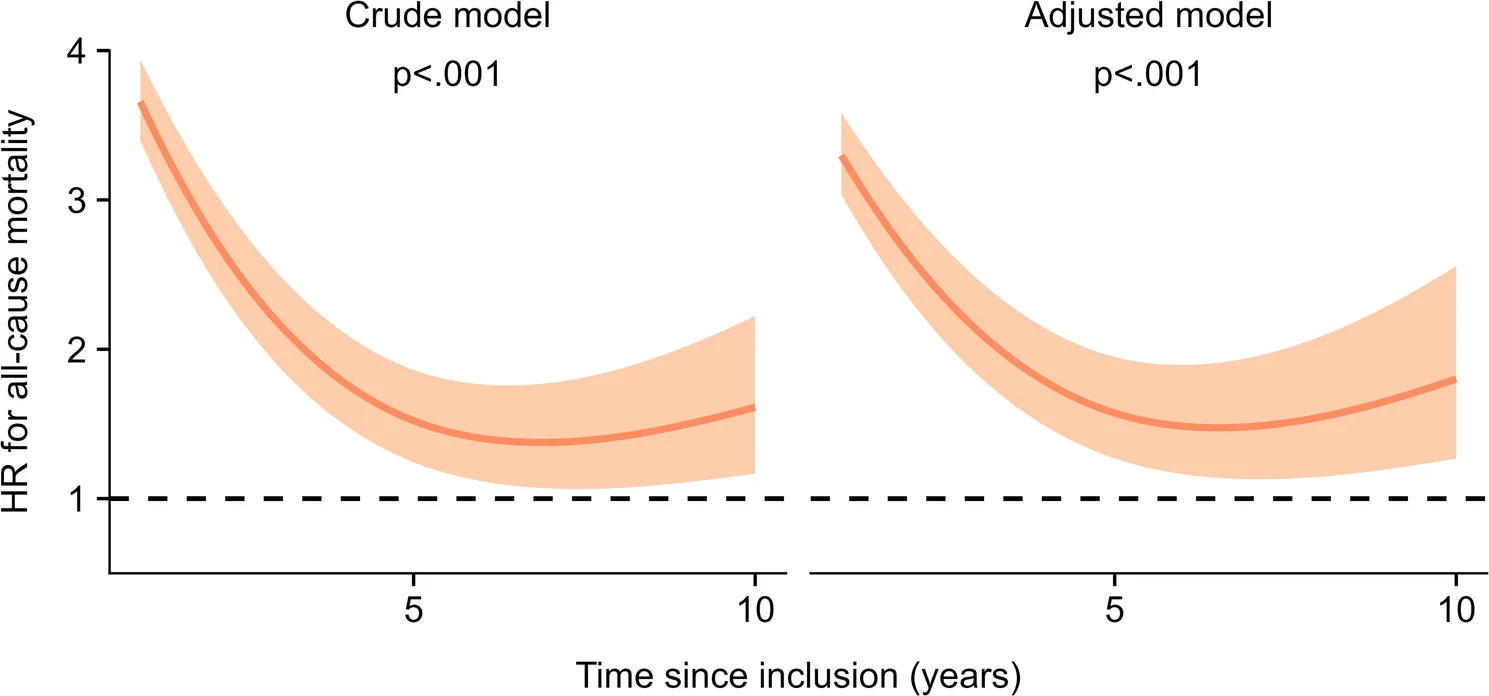
Time-dependent hazard ratios (HRs) for all-cause mortality in individuals with type 2 diabetes and any lower-extremity amputation compared with matched controls. Curves represent HRs estimated using Cox models with time-varying coefficients, modelled with natural cubic splines; shaded areas indicate 95% confidence intervals. Results are shown for crude (left) and adjusted (right) models. Overall test for time dependence, *P* < 0.001

In time-dependent analyses, the hazard of all-cause mortality following amputation was highest during the first year after amputation and declined over time, while remaining significantly elevated throughout the follow-up period (Fig. 3). The aHR at one year was 3.30 (95% CI 3.04–3.58), decreasing to 1.57 (95% CI 1.27–1.95) at five years and 1.80 (95% CI 1.27–2.56) at ten years (*P* < 0.001 for time interaction). Similar time-dependent effects were observed for cardiovascular mortality (*P<*0.001), whereas no significant temporal variation was detected for heart failure (*P* = 0.12), myocardial infarction (*P* = 0.43) or stroke (*P* = 0.074) (Supplementary Table S2).

## Discussion

### Principal findings

In this nationwide cohort of individuals with type 2 diabetes, lower-extremity amputation was associated with markedly increased risks of all-cause and cardiovascular mortality, as well as myocardial infarction, heart failure, and stroke, compared with matched controls. The excess risk was greatest during the first year following amputation and, although declining over time, remained significantly elevated throughout long-term follow-up. Major amputation was associated with higher mortality than minor amputation.

### Earlier studies

Our findings are consistent with previous studies demonstrating high mortality and adverse outcomes following diabetic foot ulcers and lower-extremity amputation. In a prospective single-centre study, the one-year outcomes following diabetic foot ulcer included a healing rate of 67%, a major amputation rate of 10%, a minor amputation rate of 19%, and a mortality rate of 9% [24]. In a 10-year retrospective cohort study of individuals with type 2 diabetes and diabetic foot ulcers, 9.9% underwent minor amputation, 3.6% experienced major amputation, and 17% died during follow-up [25]. The lifetime risk of developing a diabetic foot ulcer has been estimated at 19–34%, with approximately 20% of affected individuals undergoing lower-extremity amputation and five-year mortality rates ranging from 50% to 70% [4]. Mortality following amputation due to diabetic foot disease remains alarmingly high, with reported five-year mortality rates exceeding 70% after major amputation [6].

Our study adds to previous research by providing an updated and comprehensive population-based assessment of long-term mortality and cardiovascular outcomes following lower-extremity amputation in individuals with type 2 diabetes. While earlier studies have established an increased mortality risk, we extend these findings by illustrating how this risk varies over time, being highest during the first year after amputation and declining thereafter.

### Clinical implications

If diabetic foot ulcers occur, effective wound care, infection prevention, offloading, and optimisation of supportive treatment are essential to prevent amputation. Beyond local wound management, our findings indicate that individuals who undergo amputation face a markedly elevated risk of cardiovascular disease and mortality. This underscores the need for intensified cardiovascular risk management in this population. In clinical practice, this may include systemic cardiovascular monitoring through regular ECG assessment, echocardiography, and measurement of biomarkers of cardiac function, alongside established measures such as blood pressure, lipids, and glycaemic control, smoking cessation, and renal function monitoring and access to preventive cardiovascular glucose-lowering agents and anticoagulants in accordance with guidelines [26]. Coronary artery disease is more often silent in the presence of diabetes and novel imaging screening tools might in the future be of importance, but the latest guidelines do not recommend screening for CAD in asymptomatic patients. Strengthened follow-up using a multidisciplinary approach is therefore critical both to prevent further amputations and to improve long-term cardiovascular outcomes.

Amputation in type 2 diabetes represents a failure of preventive medicine [4] and is generally not a life-saving intervention per se, but rather a last-resort procedure for severe ischaemic or infected diabetic foot complications that fail to respond to conservative management. Our findings support the importance of preventive strategies aimed at preserving limb integrity through optimal management of diabetes, vascular disease, and foot complications [16–20, 27, 28]. In the current study, individuals who underwent amputation had a higher prevalence of peripheral arterial disease at baseline than matched controls. This likely reflects a greater burden of systemic atherosclerotic disease and may partly explain the increased cardiovascular risk associated with amputation. The excess risk remained after adjustment for established cardiovascular risk factors, suggesting that conventional risk markers do not fully capture the underlying vascular disease burden.

### Strengths and limitations

A major strength of this study is its nationwide, population-based design, utilising data from the Swedish NDR with near-complete coverage of individuals with type 2 diabetes across Sweden over a comparatively recent period. This extensive coverage ensures high external validity and enhances the generalisability of the findings. The NDR provides detailed information on cardiovascular risk factors, including HbA1c, diabetes duration, blood lipids, blood pressure, smoking status, and renal function, allowing comprehensive adjustment for potential confounders. The validity of amputation codes in the NPR has, to our knowledge, not been specifically evaluated; however, studies assessing the validity of surgical procedures in the NPR have demonstrated a mean positive predictive value of 97% [29]. Nevertheless, registry-based studies are inherently susceptible to missing data, potential inaccuracies in recorded information, and residual confounding. Furthermore, the study period partly predates the widespread clinical use of glucose-lowering agents such as glucagon-like peptide-1 receptor agonists and sodium-glucose transporter-2 inhibitors, which have demonstrated cardioprotective and renoprotective effects and may influence outcome patterns in contemporary clinical practice. It should also be noted that the current study mainly represents individuals undergoing amputation who were free from previously diagnosed severe cardiovascular disease. Consequently, the findings may not be fully representative of the broader population undergoing amputation. In addition, comprehensive data on neuropathy were not available in the present study.

## Conclusion

Lower-extremity amputation in individuals with type 2 diabetes was associated with substantially increased risks of cardiovascular events and mortality. These findings underscore the importance of optimised cardiovascular and metabolic risk management both before and after amputation, including strict glycaemic control, vascular protection, renal support, and close clinical monitoring to reduce complications and improve patient outcomes.

## Supplementary Information

Below is the link to the electronic supplementary material.

Supplementary Material 1

## Data Availability

The data were used under licence for the current research and are not publicly available. Due to participant confidentiality and restrictions under the European General Data Protection Regulation (GDPR), the data cannot be openly shared. De-identified data underlying the findings may be made available from the corresponding author upon reasonable request and submission of a written proposal, subject to relevant ethical, legal, and regulatory approvals.
