# Supplementary material for: Excess risk of cardiovascular disease and mortality after amputation in type 2 diabetes: a nationwide population study from the Swedish National Diabetes Register
Source: Cardiovasc Diabetol. 2026 Aug 24;25:260. doi: 10.1186/s12933-026-03319-5 (PMC13520145; doi:10.1186/s12933-026-03319-5)
Supplement: Supplementary file 1 [file 12933_2026_3319_MOESM1_ESM.docx]

This supplement has been provided by the authors to give readers additional information about their work. Supplement to

**Excess risk of cardiovascular disease and mortality after amputation in type 2 diabetes: a nationwide population study from the Swedish National Diabetes Register**

Authors: Karin Bergqvist^1^, Henrik Imberg^2,3^, Sara Hallström^2,4^, Jens Michelsen^2,3^, Hanna Liljebäck^2,5^, Stefan Franzén^6,7^, Anna Norhammar^8^, Annika Rosengren^2, 4^, Marcus Lind^2,4,9^


1. Department of Infectious Diseases, Institute of Biomedicine, University of Gothenburg, Gothenburg, Sweden

2. Department of Molecular and Clinical Medicine, Institute of Medicine, University of Gothenburg, Gothenburg, Sweden

3. Statistiska Konsultgruppen Sweden AB, Gothenburg, Sweden

4. Department of Medicine, Geriatrics and Emergency care, Sahlgrenska University Hospital, Gothenburg, Region Västra Götaland, Sweden

5. Department of Medicine, Skaraborg Hospital, Skövde, Sweden

6. School of Public Health and Community Medicine, Institute of Medicine, Sahlgrenska Academy, University of Gothenburg, Gothenburg, Sweden

7. BPM Evidence Statistics, Medical Evidence, BioPharmaceuticals Medical, AstraZeneca, Gothenburg, Sweden

8. Division of Cardiology, Department of Medicine Solna, Karolinska Institutet and Capio S:t Görans Hospital, Stockholm, Sweden

9. Department of Medicine, NU-Hospital Group, Trollhättan and Uddevalla, Sweden

**Contents**

**Supplementary Table S1.** Baseline characteristics of individuals with type 2 diabetes and any, major, or minor lower-extremity amputation compared with age- and sex-matched controls..................................................................................................................................3

**Supplementary Table S2.** Time-dependent hazard ratios (HRs) for all-cause and cardiovascular outcomes in individuals with type 2 diabetes with any lower-extremity amputation compared with matched controls.........................................................................5

**Supplementary Figure S1**. Flow chart of cohort selection…………………………….…..6

Supplementary Table S1. Baseline characteristics of individuals with type 2 diabetes and any, major, or minor lower-extremity amputation compared with age- and sex-matched controls.

|  | Cohort 1: Any amputation | | Cohort 2: Major amputation | | Cohort 3: Minor amputation | |
| --- | --- | --- | --- | --- | --- | --- |
|  | Cases  (n = 3,485) | Controls  (n = 13,940) | Cases  (n = 2,435) | Controls  (n = 9,740) | Cases  (n = 1,449) | Controls  (n = 5,796) |
| Age (years) | 74 (12) | 74 (12) | 75 (11) | 75 (11) | 71 (12) | 71 (12) |
| Female sex, n (%) | 1,218 (35%) | 4,872 (35%) | 964 (40%) | 3,856 (40%) | 353 (24%) | 1,412 (24%) |
| Diabetes duration (years) | 17 (10) | 12 (8) | 17 (10) | 12 (8) | 17 (9) | 11 (8) |
| Missing, n | 992 | 4,318 | 727 | 3,032 | 351 | 1,830 |
| Body mass index (kg/m²) | 28.4 (5.8) | 28.7 (5.5) | 28.0 (5.7) | 28.7 (6.6) | 29.2 (5.9) | 29.1 (5.5) |
| *Missing, n* | *965* | *4,176* | *703* | *2,929* | *350* | *1,787* |
| Smoking, n (%) | 463 (18%) | 1,014 (10%) | 354 (20%) | 619 (9.0%) | 167 (15%) | 472 (12%) |
| *Missing, n* | *911* | *4,043* | *655* | *2,841* | *335* | *1,731* |
| Systolic blood pressure (mmHg) | 138 (19) | 137 (16) | 138 (20) | 137 (16) | 138 (18) | 136 (15) |
| *Missing, n* | *879* | *3,927* | *633* | *2,760* | *321* | *1,694* |
| Diastolic blood pressure (mmHg) | 74 (11) | 75 (9) | 73 (11) | 75 (10) | 75 (11) | 76 (9) |
| *Missing, n* | *879* | *3,927* | *633* | *2,760* | *321* | *1,694* |
| HbA1c (mmol/mol) | 63 (19) | 54 (13) | 62 (18) | 54 (13) | 66 (20) | 54 (13) |
| HbA1c (%) | 7.9 (1.7) | 7.1 (1.2) | 7.8 (1.6) | 7.1 (1.2) | 8.2 (1.8) | 7.1 (1.2) |
| *Missing, n* | *878* | *3,913* | *631* | *2,753* | *321* | *1,683* |
| Peripheral arterial disease, n (%) | 2,157 (62%) | 773 (5.5%) | 1,609 (66%) | 574 (5.9%) | 831 (57%) | 303 (5.2%) |
| Microalbuminuria, n (%) | 735 (30%) | 1,744 (18%) | 502 (30%) | 1,238 (19%) | 331 (31%) | 676 (17%) |
| Macroalbuminuria, n (%) | 452 (18%) | 588 (6.2%) | 317 (19%) | 438 (6.6%) | 195 (18%) | 225 (5.8%) |
| *Missing, n* | *1,038* | *4,494* | *755* | *3,128* | *375* | *1,928* |
| eGFR (mL/min/1.73 m²) | 71 (26) | 77 (21) | 70 (27) | 75 (21) | 72 (25) | 79 (21) |
| *Missing, n* | *900* | *3,984* | *647* | *2,799* | *329* | *1,714* |
| HDL cholesterol (mmol/L) | 1.27 (0.44) | 1.31 (0.40) | 1.27 (0.44) | 1.31 (0.40) | 1.24 (0.41) | 1.28 (0.39) |
| *Missing, n* | *1,097* | *4,654* | *800* | *3,277* | *394* | *1,966* |
| LDL cholesterol (mmol/L) | 2.57 (0.99) | 2.61 (0.91) | 2.55 (1.01) | 2.61 (0.91) | 2.59 (0.98) | 2.57 (0.89) |
| *Missing, n* | *1,098* | *4,624* | *798* | *3,255* | *400* | *1,958* |
| Data are presented as mean (SD) or number (%). The number of missing values is shown for each variable, if applicable.  Age was defined as age at amputation for individuals undergoing amputation and at the corresponding index date for controls. Peripheral arterial disease was identified from ICD-10 codes I70.2 and I73.9 recorded in the Swedish National Patient Register before the date of amputation/index date. All other variables were based on the most recent registration in the Swedish National Diabetes Register prior to the amputation or index date. The mean (SD) interval between registration and amputation/index date was 1.0 (1.4) years in the amputation group and 0.9 (1.3) years in controls for the any-amputation cohort; 1.0 (1.4) and 0.9 (1.4) years, respectively, for the major amputation cohort; and 0.8 (1.3) years in both groups for the minor amputation cohort. | | | | | | |
| **Abbreviations**: eGFR, estimated glomerular filtration rate; HbA1c, glycated haemoglobin; HDL, high-density lipoprotein; LDL, low-density lipoprotein. | | | | | | |

Supplementary Table S2. Time-dependent hazard ratios (HRs) for all-cause and cardiovascular outcomes in individuals with type 2 diabetes with any lower-extremity amputation (n = 3,485) compared with matched controls (n = 13,940).

| Outcome /  Follow-up | Unadjusted HR (95% CI) | *P* | Adjusted HR (95% CI) | *P* |
| --- | --- | --- | --- | --- |
| All-cause mortality |  | <0.001 |  | <0.001 |
| 1 year | 3.66 (3.40, 3.94) |  | 3.30 (3.04, 3.58) |  |
| 5 years | 1.52 (1.24, 1.86) |  | 1.57 (1.27, 1.95) |  |
| 10 years | 1.61 (1.17, 2.22) |  | 1.80 (1.27, 2.56) |  |
| Cardiovascular mortality |  | <0.001 |  | <0.001 |
| 1 year | 3.46 (3.00, 3.99) |  | 2.97 (2.55, 3.46) |  |
| 5 years | 1.93 (1.32, 2.82) |  | 1.88 (1.26, 2.78) |  |
| 10 years | 2.03 (1.15, 3.58) |  | 2.14 (1.18, 3.89) |  |
| Heart failure |  | 0.007 |  | 0.12 |
| 1 year | 2.87 (2.55, 3.23) |  | 2.31 (2.03, 2.63) |  |
| 5 years | 2.10 (1.51, 2.92) |  | 1.86 (1.32, 2.62) |  |
| 10 years | 2.00 (1.14, 3.50) |  | 1.89 (1.08, 3.30) |  |
| Myocardial infarction |  | 0.47 |  | 0.43 |
| 1 year | 2.00 (1.68, 2.39) |  | 1.69 (1.40, 2.04) |  |
| 5 years | 2.29 (1.42, 3.70) |  | 2.03 (1.24, 3.32) |  |
| 10 years | 1.59 (0.71, 3.56) |  | 1.51 (0.67, 3.41) |  |
| Stroke |  | 0.066 |  | 0.074 |
| 1 year | 1.78 (1.49, 2.13) |  | 1.63 (1.34, 1.97) |  |
| 5 years | 1.28 (0.78, 2.11) |  | 1.25 (0.75, 2.09) |  |
| 10 years | 2.11 (1.01, 4.40) |  | 2.23 (1.06, 4.69) |  |
| Hazard ratios (HRs) and 95% confidence intervals (CIs) were estimated using Cox regression with time-varying coefficients modelled by natural cubic splines.  Adjusted models included age, sex, diabetes duration, BMI, blood pressure, smoking status, albuminuria, eGFR, LDL and HDL cholesterol at baseline.  *P*-values correspond to the overall test for time interaction.  **Abbreviations:** BMI, body mass index; CI, confidence interval; DBP, diastolic blood pressure; eGFR, estimated glomerular filtration rate; HDL, high-density lipoprotein; HR, hazard ratio; LDL, low-density lipoprotein; SBP, systolic blood pressure. | | | | |

**Supplementary Figure S1.** Flow chart of cohort selection. Individuals with type 2 diabetes undergoing a first lower-extremity amputation were identified from the Swedish National Diabetes Register. Cases with prior stroke, myocardial infarction, heart failure, death on the amputation date, or no prior NDR registration were excluded. For each case, four controls were selected using risk-set sampling and matched on age, sex, and calendar time. Controls were required to be alive and free from prior amputation, myocardial infarction, heart failure, and stroke at the index date.
